# Salvage surgery and conversion surgery for patients with nonsmall cell lung cancer: a narrative review

**DOI:** 10.1097/JS9.0000000000001921

**Published:** 2024-07-11

**Authors:** Hao-Ji Yan, Xiang-Yun Zheng, Ya-Ting Zeng, Jia-Xin Wan, Jing Chen, Zhi-Qiang Deng, Yu-Yang Mao, Wen-Long Hu, Jun-Jie Zhang, Ai-Ling Zhong, Chun-Yan Zhao, Wen-Jun Mao, Dong Tian

**Affiliations:** a Department of Thoracic Surgery, West China Hospital, Sichuan University, Chengdu, People's Republic of China; b Department of General Thoracic Surgery, Juntendo University School of Medicine, Tokyo, Japan; c School of Clinical Medicine, North Sichuan Medical College; d School of Medical Imaging, North Sichuan Medical College, Nanchong; e Department of Nuclear Medicine, West China Hospital, Sichuan University, Chengdu; f Department of Thoracic Surgery, The Affiliated Wuxi People's Hospital of Nanjing Medical University, Wuxi, People's Republic of China

**Keywords:** conversion surgery, immunotherapy, nonsmall cell lung cancer, salvage surgery, sublobar resection, targeted therapy

## Abstract

Nonsmall cell lung cancer (NSCLC) remains the leading cause of cancer-related deaths. With the development of screening, patient selection, and treatment strategies, patients’ survival outcomes and living quality significantly improved. However, some patients still have local recurrence or residual tumors after receiving definitive therapies. Salvage surgery has been regarded as an effective option for recurrent or residual NSCLC, but its effectiveness remains undetermined. Furthermore, conversion surgery is a special type of salvage surgery for tumors converted from ‘initially unresectable’ to ‘potentially resectable’ status due to a favorable response to systemic treatments. Although conversion surgery is a promising curative procedure for advanced NSCLC, its concept and clinical value remain unfamiliar to clinicians. In this narrative review, we provided an overview of the safety and efficacy of salvage surgery, especially salvage surgery after sublobar resection in early-stage NSCLC. More importantly, we highlighted the concept and value of conversion surgery after systemic treatment in advanced NSCLC to gain some insights into its role in the treatment of lung cancer.

## Introduction

Highlights

- Conversion surgery has the potential to improve the prognosis of patients with nonsmall cell lung cancer (NSCLC) after systemic treatment if downstaging is achieved.
- Salvage surgery is the primary treatment for patients with local recurrence (LR) after sublobar resection.
- Salvage surgery for LR after stereotactic body radiotherapy (SBRT) in selected patients with operable NSCLC is safe and feasible, which may be superior to other salvage treatments.
- Salvage surgery after systematic treatment is promising, with an acceptable long-term survival outcome in strictly selected patients.

NSCLC is one of the most common malignant tumors worldwide^1^. Much progress has been made recently for NSCLC, such as screening, patient selection, and treatment strategy. Currently, there are various therapies for NSCLC patients with different stages, including surgical resection, SBRT, chemoradiotherapy (CRT), targeted therapy, and immunotherapy^2^. Lobectomy has been considered the standard treatment for patients with resectable early-stage NSCLC^3^. Meanwhile, increasing evidence has shown that sublobar resection can achieve comparable overall survival (OS) to lobectomy^4,5^. However, it is reported to be associated with a higher LR. SBRT is a recommended option for early-stage NSCLC patients at high or prohibitive risk for surgery, including poor lung function and severe comorbidities. Its effectiveness has been confirmed by several clinical trials, with 3-year local control rates of about 90%^6–10^. Once the tumor develops to an advanced and unresectable stage, definitive CRT is a common therapy modality for patients with NSCLC. In recent years, targeted therapy and immunotherapy have brought conspicuous survival benefits for patients with advanced NSCLC^11,12^. Immunotherapy, at the forefront of treatment in oncogenic driver-negative NSCLC, has been increasingly used and continues to demonstrate a significant survival benefit^13^. However, despite the progress of these therapies, many patients still experience recurrent or residual. The rate of LR after SBRT for early-stage NSCLC ranges from 5 to 20%^14,15^, and up to 35% of patients will experience LR or persistent diseases after definite CRT for advanced NSCLC^16^. Additionally, most patients with advanced NSCLC inevitably develop acquired resistance to targeted therapy and immunotherapy, leading to recurrent and residual tumors commonly^17,18^. Salvage surgery has been regarded as an available therapy for these NSCLC patients. Salvage surgery can be defined into two categories: first, true salvage surgery, which is surgery for LR or residual tumor after definitive therapies and second, conversion surgery, which is surgery for tumors that have converted from an ‘initially unresectable’ to a ‘potentially resectable’ status and were not initially planned. Conversion surgery is a promising procedure for improving survival outcomes, but it is unfamiliar to clinicians, resulting in rare reports and attention. In this narrative review, we overviewed the effectiveness of salvage surgery (Table 1) and conversion surgery for NSCLC after different definitive therapies. We reviewed the clinical outcomes of patients undergoing salvage surgery for NSCLC patients and which patients are likely to benefit from salvage surgery. More importantly, we discussed the concept and clinical value of conversion surgery for advanced NSCLC to gain more attention from clinicians.

**Table 1:** Summary of studies reporting salvage surgery after different definitive therapies.

Salvage surgery after definitive therapies	Author (year)	Number of patients	Interval to salvage surgery (months) (range)	R0/R1/R2	Type of resection						OS (median, months)	RFS (median, months)	Complication
					S	W	CP	L	BL	SL			
Lung resection													
	Dolan *et al*.^32^	23	34	NR	3	20	—	—	—	—	5-year 80%	3-year 41% (DFS) 5-year 9% (DFS)	35% (8/23)
	Kasprzyk *et al*.^35^	21	9	20/1/0	—	—	21	—	—	—	27	9	NR
	Cardillo *et al*.^80^	165	42 (4—192)	NR	—	—	165	—	—	—	5-year 38%	NR	55% (91/165)
	Chataigner *et al*.^81^	17	NR	17	—	—	17	—	—	—	5-year 41%	NR	NR
	Jungraithmayr *et al*.^82^	26	NR	26	—	—	26	—	—	—	5-year 23%	NR	29%
SBRT													
	Verstegen *et al*.^43^	9	22 (10—35)	8/1/0	—	1	1	6	—	1	NR	NR	33% (3/9)
	Antonoff *et al*.^44^	21	16 (6—72)	NR	8	—	1	12	—	—	47 3-year 53%	19.2 (DFS)	19% (7/21)
	Hamaji *et al*.^45^	12	NR	NR	2	1	—	9	—	—	5-year 80%	NR	25% (3/12)
	Yamanashi *et al*.^46^	12	NR	12/0/0	—	—	—	—	—	—	5-year 58% 10-year 33%	5-year 42% (PFS) 10-year 33% (PFS)	NR
Definitive CRT													
	Suzuki *et al*.^24^	46	0 (0—4)	41/NR	2	5	2	37	—	—	2-year 79% 5-year 66%	2-year 30% (PFS) 5-year 30% (PFS)	24% (11/46)
	White *et al*.^36^	28	34	NR	—	—	28	—	—	—	5-year 43%	NR	46% (13/28)
	Yang *et al*.^51^	31	4 (2—26)	30/1/0	—	—	—	30	1	—	33 1-year 77% 2-year 53% 3-year 42% 5-year 31%	10.3 1-year 42% 2-year 30% 5-year 23%	48% (15/31)
	Shimizu *et al*.^54^	110	3 (0—150)	NR	2	4	18	78	8	—	3-year 67%	3-year 49.8%	18% (grade 3—5)
	Schreiner *et al*.^59^	13	7 (3—39)	11/2/0	3	1	1	6	2	—	30 3-year 46% 5-year 46%	21.9 5-year 44%	46% (6/13)
	Bograd *et al*.^62^	30	9 (IQR, 6—16)	24/6/0	—	—	7	22	1	—	24	NR	57% (17/30)
	Casiraghi *et al*.^83^	35	7 (1—39)	27/2/6	—	—	17	11	1	—	13 2-year 38% 3-year 32% 5-year 20%	12 (DFS) 2-year 25% (DFS) 3-year 20% (DFS) 5-year 20% (DFS)	26% (major 9) 26% (minor 9)
	Sawada *et al*.^84^	8	9 (3—50)	8/0/0	—	—	1	7	—	—	5-year 75%	NR	38% (3/8)
	Kuzmik *et al*.^85^	14	NR	NR	2	1	2	8	—	1	2-year 49%	33 (DFS)	43% (6/14)
	Bauman *et al*.^86^	24	5 (1—22)	23/2/0	—	1	10	10	4	—	30 3-year 47%	12 (PFS)	58% (14/24)
	Joosten *et al*.^87^	30	NR	23/6/1	—	—	13	17	—	—	21 1-year 67% 2-year 50% 3-year 47%	8 (DFS)	47% (14/30)
	Shimada *et al*.^88^	18	9 (1—66)	16/2/0	—	—	5	13	—	—	3-year 78%	3-year 72%	28% (5/18)
	Romero-Vielva *et al*.^89^	27	NR	27/0/0	—	—	13	7	7	—	76 1-year 74% 3-year 58% 5-year 53%	15	19% (5/27)
	Watanabe *et al*.^90^	27	9 (1—92)	24/1/2	—	—	9	18	—	—	5-year 63%	5-year 27%	59% (16/27)
	Kobayashi *et al*.^91^	38	22 (2—168)	35/3/0	4	—	8	24	2	—	5-year 41%	5-year 44% [PFS]	15% (3/38)
	Dickhoff *et al*.^92^	15	5 (1—22)	NR	1	—	8	4	2	—	46	44 (EFS)	40% (6/15)
	Kobayashi *et al*.^93^	23	41 (3—170)	22/0/1	2	—	7	13	1	—	5-year 45%	5-year 42.2% (PFS)	4% (1/23)
	Kaba *et al*.^94^	30	NR	28/NR	5	—	8	14	2	—	19±13 (mean±SD)	14±12 (mean±SD)	60% (18/30)
	Ye *et al*.^95^	126	NR	NR	NR	NR	NR	NR	NR	NR	3-year 52% 5-year 36%	NR	NR
	Moore *et al*.^96^	12	NR	NR	—	4	1	7	—	—	38	NR	NR
Targeted therapy													
	Li *et al*.^19^	18	NR	NR	—	—	—	18	—	—	Not reached	23.4 (PFS)	6% (1/18)
	Hishida *et al*.^20^	9	NR	NR	—	—	2	6	1	—	32	6	NR
	Ohtaki *et al*.^66^	36	14 (IQR, 6—31 days)	29/6/1	3	2	1	28	1	—	3-year 75% 5-year 44%	3-year 22.2% 5-year 22.2%	6% (2/36)
	Song *et al*.^67^	9	5—14 days	9/0/0	—	—	—	9	—	—	25	14 (EFS)	11% (1/9)
Immunotherapy													
	Etienne *et al*.^21^	21	22 (IQR, 18—35)	21	3	—	1	17	—	—	NR	NR	43% (9/21)
	Higuchi *et al*.^22^	13	10 (2—40)	NR	—	—	—	12	—	—	2-year 76%	2-year 71.2% (DFS)	23% (grade 3—5)
	Ueno *et al*.^77^	11	9 (4—20)	11	—	—	—	10	1	—	NR	NR	27% (grade 3—5)
	Bott *et al*.^78^	11	3 (0—6)	10/NR	—	11	1	8	2	—	2-year 77%	1-year 58% (DFS) 2-year 42% (DFS)	32%

BL, bilobectomy; CP, completion pneumonectomy; CRT, chemoradiotherapy; DFS, disease-free survival; EFS, event-free survival; IQR, interquartile range; L, lobectomy; NR, not reported; OS, overall survival; PFS, progression-free survival; RFS, recurrence-free survival; S, segmentectomy; SBRT, stereotactic body radiation therapy; SD, standard deviation; SL, sleeve-lobectomy; W, wedge resection.

## Conversion surgery

**Figure 1:**
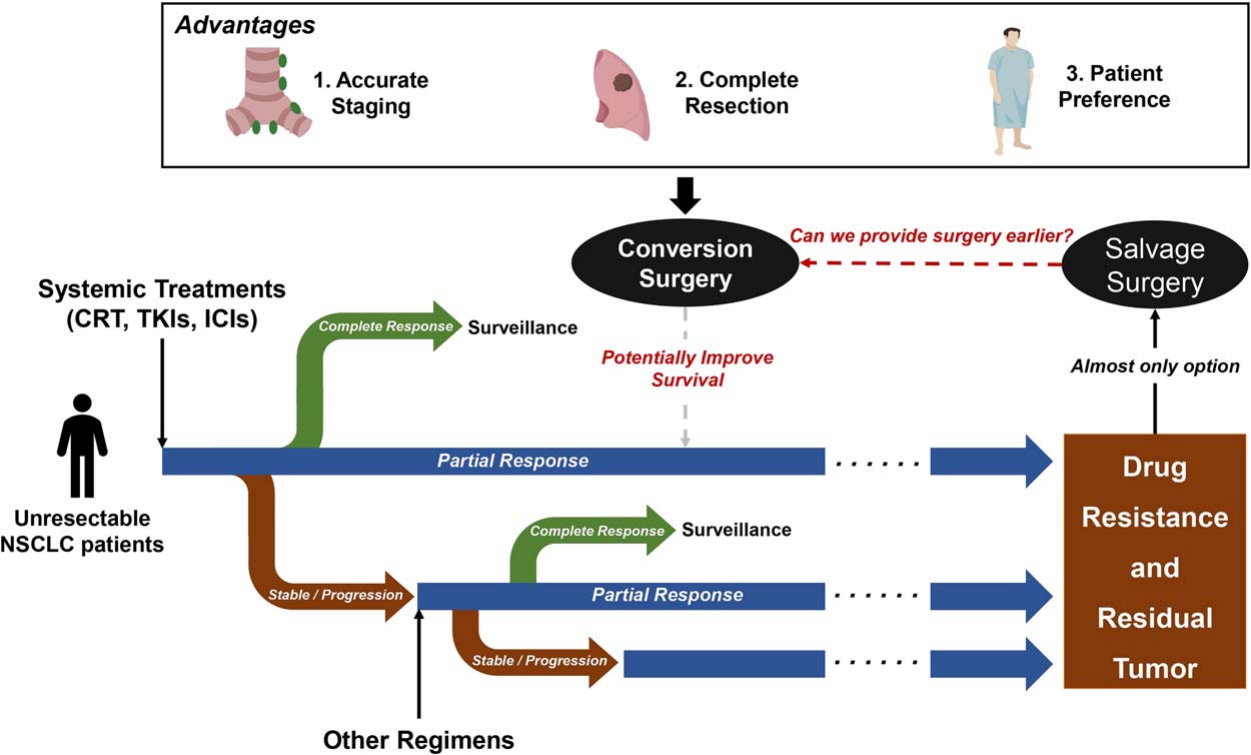
The treatment strategies for unresectable NSCLC patients. If patients fail to achieve a complete response after systemic treatments, salvage surgery is almost the only treatment option. Conversion surgery may provide survival benefits for these patients. CRT, chemoradiation treatment; ICIs, immune checkpoint inhibitors; NSCLC, nonsmall cell lung cancer; TKIs, tyrosine kinase inhibitors.

Systemic treatments are undoubtedly the primary treatments for unresectable NSCLC patients. However, if patients fail to achieve a complete response in the long-term treatment, drug resistance is usually inevitable and causes tumor residual or progression. Salvage surgery is almost the only curative-intent option for those patients. Considering this situation, can we provide surgery earlier to achieve better tumor control and improve survival outcomes (Fig. 1)?

In recent years, the development of targeted therapy and immunotherapy provided the possibility for conversion surgery after downstaging^11,12^. Conversion surgery is defined as the surgery for tumors that have converted from an ‘initially unresectable’ to a ‘potentially resectable’ status after definitive systemic treatments. The patient may not require finishing definitive systemic treatments; instead, a dynamic assessment throughout the treatment process may be more appropriate. It should be emphasized that conversion surgery is different from surgery after neoadjuvant/induction therapy for NSCLC patients. The most significant difference is that the conversion surgery is initially unplanned and is performed when tumor downstaging is achieved. In other words, surgery after neoadjuvant/induction therapy is usually for initially operable NSCLC, but conversion surgery is for initially inoperable NSCLC. Although the neoadjuvant/induction therapy may be applied for initially inoperable NSCLC in a few cases, initially inoperable patients should be separately analyzed. In addition, the term ‘conversion surgery’ is not commonly used in the field of lung cancer and is often confused with true salvage surgery. It should be independently introduced in the future due to its growing significance. Conversion surgery as an additional treatment has three advantages over systemic treatment alone. First, conversion surgery can provide accurate staging to guide further systemic treatment. After conversion lung resection with systematic lymph node dissection, the pathological stage can be acquired to assess the response of the tumor, which is valuable to subsequent treatment. Second, conversion surgery can provide complete resection for patients with advanced NSCLC. The total amount of cancer in the body will be significantly reduced, and the patient’s prognosis will be improved by radically resecting the primary tumor and lymph nodes. Third, surgery is a personal preference of some advanced NSCLC patients. Some initially unresectable NSCLC patients expect surgery after systemic treatment to remove tumors from their bodies, although it is usually not recommended in current guidelines.

Previous studies have shown that if downstaging was achieved after systemic treatment, conversion surgery may improve survival outcomes for advanced NSCLC^19–24^. A study reviewed initially inoperable NSCLC patients (IIIB–IV) who achieved tumor downstaging (≤IIIA) after targeted treatment and showed that the median progression-free survival (PFS) of the targeted therapy plus conversion surgery group (23.4 vs. 12.9 months) was significantly longer than that of the targeted therapy alone^19^. Notably, all patients in the conversion surgery group achieved R0 resection. However, another study reported that conversion surgery had no obvious survival benefit because recurrence-free survival (RFS) was only 6 months^20^. The reasons for the difference in results may be firstly, no adequate preoperative examination. No positron emission tomography-computed tomography examination was performed for more accurate staging in the latter study. Most patients (7/9, 77.8%) showed a more advanced pathologic stage than their preoperative clinical stage. In the former study^19^, a few patients (4/18, 22.2%) were in a later stage compared with the clinical stage. Second, treatment interval. The interval to conversion surgery was 17 months (2–36 months) in the latter study, while the mean duration between targeted therapy and conversion surgery was 4 months (2–12 months) in the former study. Prolonged duration can increase the risk of drug resistance and further affect PFS. However, it should be noted that in the study of Li *et al*.^19^, it seems unreasonable to compare the survival outcomes of the group undergoing targeted therapy plus conversion surgery with the group undergoing targeted therapy alone. The patients who were able to undergo conversion surgery had already achieved downstaging (partial response). However, those who received only targeted treatment showed various responses. A comparison between targeted treatment plus conversion surgery and targeted treatment alone for downstaging patients is necessary to confirm the additional survival benefits of conversion surgery.

**Figure 2:**
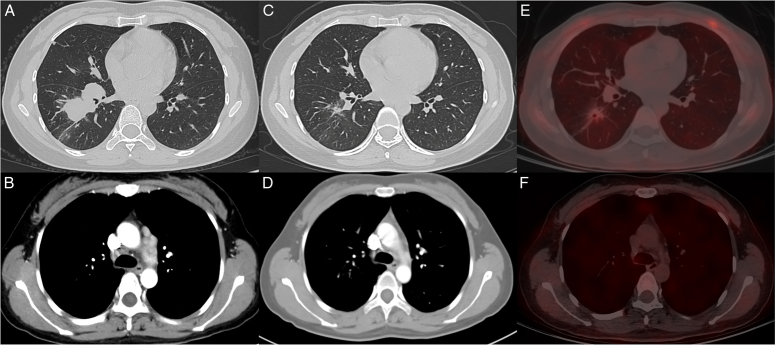
A case about conversion surgery for initially unresectable NSCLC. A 35-year-old female presented with a mass in the right lower lobe of the lung (A), accompanied by enlarged contralateral mediastinal lymph nodes (B). Preoperative biopsy confirmed the diagnosis of lung adenocarcinoma and mediastinal lymph node metastasis (cT3N3M0, stage IIIC), with an epidermal growth factor receptor exon 19 deletion. Initially, the patient received two months of treatment with almonertinib. The tumor responded well to almonertinib, with significant shrinkage of the primary lesion (C) in the right lower lobe and the disappearance of enlarged contralateral mediastinal lymph nodes (D). In the further assessment with PET/CT, the SUVmax value of the primary lesion was 1.91 (E), with no abnormal uptake in the hilar and mediastinal lymph nodes (F). Subsequently, the patient underwent right lower lobe resection and systematic lymph node dissection, with postoperative pathological staging revealing ypT1cN2M0, stage IIIA. Postoperatively, the patient continued to receive almonertinib therapy. Up to now, the patient has been followed up for 1 year and remains alive with no recurrence. NSCLC, nonsmall cell lung cancer; PET/CT, positron emission tomography/computed tomography; SUV, standardized uptake value.

Conversion surgery after immunotherapy plus chemotherapy in NSCLC patients has also been reported. Etienne *et al*.^21^ reported 21 patients with initially unresectable advanced-staged NSCLC who underwent conversion surgery after immune checkpoint inhibitors (ICIs). After a median follow-up of 16 months, 90% of patients still survived. In addition, the 2-year disease-free survival rate and 2-year OS rate reported by Higuchi *et al*.^22^ of 13 advanced NSCLC patients who underwent salvage surgery (about nine cases underwent conversion surgery) after immunotherapy were 71.2 and 76.2%, respectively. The emergence of conversion surgery brings the possibility of long-term survival for patients with advanced NSCLC after immunotherapy. Other studies also showed encouraging outcomes but did not strictly focus on conversion surgery. Sonobe *et al*.^23^ reported 19/29 patients who underwent conversion surgery after definitive CRT. The 5-year OS and RFS were 52 and 49%, respectively. In another study, among 46 patients undergoing CRT, tyrosine kinase inhibitors (TKIs), and ICIs reported by Suzuki *et al*.^24^, 37 patients with NSCLC underwent conversion surgery. The 5-year OS and PFS of all patients were 66 and 30%. However, most studies were single-arm settings and confused with true salvage surgery because the concept of conversion surgery is unfamiliar to lung cancer clinicians. More separate and comparative studies are imperative to confirm its effectiveness. In this review, we provided a conversion surgery case at our center (Fig. 2). A 35-year-old female was initially diagnosed with unresectable NSCLC (cT3N3M0) with epidermal growth factor receptor (EGFR) mutation. National Comprehensive Cancer Network (NCCN) guidelines recommended treatment for this patient, which is definitive chemoradiation followed by immunotherapy. The 1-year PFS rate is about 55.9%, as reported in the PACIFIC trial^25^. In our center, after 2 months of EGFR-TKI treatment (almonertinib), conversion surgery was provided because the tumor converted to resectable status. After conversion surgery (ypT1cN2M0), almonertinib maintenance therapy was provided. Up to now, this patient is alive without recurrence for 1 year.

The proportion of eligible patients after systemic treatments for conversion surgery is unclear. All of the patients with objective responses are potential candidates for conversion surgery, but the actual proportion of conversion surgery candidates needs further study. In addition, the timing of conversion surgery and accurate preoperative staging should be noted when deciding on conversion surgery. Although prolonged systemic treatment may cause drug resistance or progression, according to previous studies, sufficient systemic treatment could contribute to complete resection for advanced-stage NSCLC. Therefore, a suitable timing for conversion surgery is worth studying further to achieve optimal efficacy. Tumor stopping response to the systemic treatment and converting to resectable status may be a suitable timing for conversion surgery, but further study is warranted. As stated, positron emission tomography-computed tomography is highly recommended preoperatively to obtain more accurate staging for conversion surgery decisions.

## Salvage surgery after surgical lung resection

In 1995, the Lung Cancer Study Group reported the superiority of lobectomy for early-stage NSCLC, which became the standard mode from that time^26^. Subsequently, increasing studies reported the sublobar resection for early-stage NSCLC^27–29^. The Japan Clinical Oncology Group (JCOG) 0802/West Japan Oncology Group 4607L was the first randomized trial to show the superiority of segmentectomy over lobectomy in the OS (94.3 vs. 91.1%) of small-sized peripheral NSCLC^30^. At the same time, Cancer and Leukemia Group B (CALGB) 140503 also confirmed the noninferiority of sublobar resection in the western population^4^. However, the frequency of LR in sublobar resection was twice as high as that in lobectomy (10.5 vs. 5.4%) in JCOG 0802^30^. In CALGB 140503, the locoregional recurrence rate was still higher in the sublobar resection group (13.4 vs. 10.0%)^4^.

In NSCLC patients with LR after surgery, the guidelines of the 2023 NCCN advocated radical re-resection for these patients without evidence of distant disease^31^. Previous studies have reported that salvage lobectomy after sublobar resection for LR had similar 5-year OS (79.6 vs. 70.6%) and complications (34.8 vs. 34.7%) compared to initial lobectomy for stage I-II NSCLC^32^. However, there was a major problem with their study in that they calculated the survival for both salvage surgery (after initial sublobar resection) and initial lobectomy group from the time of initial surgery. This approach resulted in all patients in the salvage group having survived more than 40 months, whereas patients in the lobectomy group began to die at various times, starting from 0 months. On the other hand, many patients will convert to inoperable status after initial sublobar resection. Therefore, in these cases, the initial surgery decision should carefully consider the risk of LR and resectability for salvage surgery.

Compared with other salvage treatments, salvage surgery after initial sublobar resection is not always reported to be the optimal treatment. Dolan *et al*.^32^ reported that the survival outcomes of patients undergoing salvage surgery were worse than those of patients who underwent SBRT only after recurrence (79.6 vs. 82.5%). Whether the lobectomy is the optimal salvage treatment for patients after sublobar resection is undetermined. Although there should be selection bias, SBRT may provide noninferior outcomes, at least, which remains to be further studied.

In terms of technical difficulty, lobectomy after segmentectomy is feasible but difficult due to severe adhesions around hilar structures. It was difficult to strip the connective tissue after initial sublobar resection, making it risky for the second procedure to cause damage to the trachea and blood vessels. However, it does not mean that minimally invasive surgery cannot be performed. Evidence showed that there was no significant difference between thoracotomy and video-assisted thoracoscopic surgery in salvage lobectomy^33^.

Pneumonectomy has also been reported as a feasible salvage surgical mode for selected NSCLC patients after initial surgery. Existing studies have shown that the survival of patients undergoing completion pneumonectomy was encouraging. Miyahara *et al*.^34^ reviewed 32 pooled studies, including 1157 patients who underwent pneumonectomy for LR (464 cases) or second primary/primary (553 cases) lung cancer after the first procedure. In the LR population, pooled 3-year and 5-year OS were 47.6 and 33.8%, showing encouraging efficacy. In addition, compared with systemic treatment (CRT or chemotherapy), salvage pneumonectomy for locally recurrent NSCLC after initial surgery showed a significant improvement in early-term and long-term survival^35^. Although there were considerable postoperative complications after salvage pneumonectomy (80.9 vs. 48.3%), survival benefits were still obtained. Furthermore, White *et al*.^36^ reported that the 5-year OS survival of patients who underwent salvage pneumonectomy is comparable to those who underwent initial pneumonectomy (43.1 vs. 38.5%). Despite salvage pneumonectomy being associated with high perioperative complications, it did not threaten postoperative survival in the context of good patient general condition^36^.

## Salvage surgery after stereotactic body radiotherapy

SBRT, also known as stereotactic ablative radiotherapy, consists of three to five high doses of radiation, while the total radiation dose used is comparable to conventional radiotherapy (RT)^37,38^. Characterized by promising efficacy and acceptable toxicity rates, SBRT has been an important alternative to surgery for clinical stage I NSCLC patients who refuse or cannot tolerate surgery^6,39,40^. Its high dose makes SBRT more biologically potent, resulting in high rates of local tumor control^39^. The efficacy of SBRT for mostly inoperable stage I NSCLC has been reported by several prospective clinical trials with 3-year local control rates ranging from 80 to 95%, achieving a comparable clinical efficacy to lobectomy for early-stage NSCLC^8–10^. Previous studies also have shown a high rate of primary tumor control of SBRT in patients with operable NSCLC^41,42^. Although SBRT was associated with favorable survival outcomes for early-stage NSCLC, high LR rates (5-year LR rate ranging from 10 to 30%) were significant issues for SBRT.

Increasing evidence indicated that salvage surgery for selected NSCLC patients with LR following SBRT is safe and feasible. According to Verstegen *et al*.^43^ report, the 5-year OS in patients who underwent salvage surgery for LR after SBTR was 80%. Similarly, an analysis that included 37 patients from four institutions showed that the median OS of those patients was 46.9 months, and the 3-year survival was 71.8%^44^. Compared with other nonsurgical salvage therapies, Hamaji *et al*.^45^ reported that salvage surgery had a better therapeutic effect for clinical stage I NSCLC patients, with 5-year OS reaching 79.5%. By extending the follow-up time, the updated 5-year OS was 58.3% in the same patient cohort, lower than that reported in other salvage resection series^46^. The difference might be associated with comorbidities and the large proportion of the elderly (the median age of 76) in the patients’ cohort, with 50% of patients in the study dying of noncancer-related causes. Notably, a prospective study showed that the 5-year local control rate of salvage SBRT for NSCLC patients with LR was 94.8%^47^. However, the failure of initial SBRT indicated that the tumor was resistant to radiation, and repeated SBRT might no longer be applicable. Since most recurrences after isolated LR were nondisseminated locoregional failures, removing tumor tissues and evaluating regional lymph nodes by salvage surgery is critical for potentially preventing further recurrences and guiding adjuvant therapy.

Although salvage surgery appears to be an effective option for patients with LR after SBRT, there are still some unsolved problems. Firstly, only a few patients who underwent SBRT were considered operable before treatment due to worse general conditions. Most patients may still be inoperable when the LR occurs. Secondly, surgical problems that may be encountered during the operation are a high incidence of intrapleural adhesion (about 20%^45,48^) and fibrosis (about 50%^43,49^) after SBRT. Intrapleural adhesion and fibrosis complicate salvage surgery and become the main technical problems^45^.

## Salvage surgery after definitive chemoradiotherapy

Previous studies have shown that approximately 30% of NSCLC patients are diagnosed with locally advanced NSCLC, and most of them have lost the opportunity for surgical treatment at the time of diagnosis^50^. For such patients with inoperable NSCLC, the definitive concurrent chemoradiation is regarded as an effective treatment^31^. However, 5-year survival rates of patients with advanced NSCLC receiving definitive CRT are only about 30%^51^. A high rate of local residual or recurrence in patients with unresectable NSCLC after definitive CRT was usually reported^50,52^.

Previous reports have emphasized that salvage surgery is feasible, with acceptable perioperative mortality and the possibility of long-term survival in strictly selected patients. Hamada *et al*.^53^ reviewed nine pooled studies with a total of 200 patients with initially unresectable NSCLC who underwent salvage surgery after definitive CRT. The median OS and RFS after salvage surgery were 13–76 months and 10–22 months, respectively. Moreover, the median morbidity was 41% (range, 15–62%), and the weighted average mortality was 4% (range, 0–11%), which appeared acceptable. Another study reported excellent survival outcomes with a low rate of mortality (30-day and 90-day mortality, 0 and 0.9%, respectively) for those patients^54^. In their cohort of 110 patients with a high proportion of stages III and IV, the 5-year OS and RFS were 59.8 and 46.3%, respectively. In contrast, the median OS of patients who received only chemotherapy or RT for LR after definitive CRT was 9 months^55^ and 11–15 months^56,57^, which was shorter than that reported after salvage surgery^23,24,54,58,59^. Notably, Uramoto *et al*.^60^ reported that the survival outcomes of patients with locally advanced NSCLC treated with salvage surgery were comparable to that of surgery after induction chemotherapy. The 5-year OS rates of patients undergoing surgery after induction chemotherapy and salvage surgery were 65.2 and 62.2%, respectively. This finding was encouraging, but more evidence is warranted.

The favorable prognostic factors for salvage surgery included the long interval from RT to salvage surgery, the significant radiation descent period, and R0 resection. The shorter time from initial therapy to salvage surgery may indicate that the tumor progresses rapidly and is difficult to control. As for downstaging, Romero-Vielva *et al*.^58^ showed that both T downstaging and N downstaging increased survival compared to the non-downstaging group, while the survival benefits of T downstaging were more significant. Furthermore, the completeness of resection is associated with prognosis. Patients with R0 resection could benefit from salvage surgery, while incomplete resection did not (the median OS, 60 vs. 20 months)^61,62^. Therefore, careful preoperative evaluation should be performed to assess the possibility of R0 resection.

## Salvage surgery after targeted therapy

Targeted therapy that targets specific molecular pathways has dramatically improved clinical outcomes in patients with advanced NSCLC^63^. Such as EGFR-TKIs and anaplastic lymphoma kinase-TKIs, have been successfully applied for NSCLC patients carrying driver gene alterations^64,65^. However, despite the favorable response to TKI treatment, most patients inevitably develop acquired resistance to TKIs, resulting in recurrence and residual tumors^17,18^. Salvage surgery of LR or residual tumors may help such patients to achieve long-term survival.

Salvage surgery can minimize the tumor burden of residual tumors and improve the prognosis of those patients. Ohtaki *et al*.^66^ reported 36 NSCLC patients who underwent tumor resection after EGFR or anaplastic lymphoma kinase-TKI treatment. The 3- and 5-year OS rates after the salvage surgery were 75 and 44%, respectively, which was encouraging for patients with advanced NSCLC. However, the 3-year RFS rate was just 22%^66^. It may be explained that salvage surgery after failure of targeted therapy showed a potential OS benefit by reducing the local tumor burden. Patients with advanced diseases are prone to re-relapse after surgical resection. Nevertheless, salvage surgery seems necessary because it contributes to prolonged OS. Other studies with sample size <10 also found similar results that salvage surgery can improve OS but not RFS. Song *et al*.^67^ showed that the median OS and median event-free survival of the nine patients with advanced NSCLC were 25 and 14 months, respectively. Similarly, the median RFS reported by Hishida *et al*.^68^ in 2014 (four cases) was 1.2 years, but all patients survived more than 5 years. According to a study report, the radiological responses to TKI treatment and lower carcinoembryonic antigen value may be good prognostic factors for OS, while the age and pathological T stage of initial treatment may be independent prognostic factors for RFS^66^. In terms of safety, a small number of patients had obvious fibrosis, which may be related to targeted therapy, increasing the difficulty of lymph node dissection. The significant regression of the tumor after targeted therapy may lead to the replacement of tumors with fibrous scar tissue. Even though the reported 90-day mortality rate was 0, which suggested that salvage surgery after targeted treatment is not risky to perform^66^.

## Salvage surgery after immunotherapy

The use of immunotherapy has led to improved response rates and unprecedented survival benefits in NSCLC patients^69^. The estimated 5-year OS rate of patients with advanced NSCLC treated with nivolumab was 16%, as reported in the CA209-003 study^70^, while the 5-year OS rate of patients with stage IV lung cancer in the past history was only 6%^71^. Additionally, compared with chemotherapy alone, ICIs combined with chemotherapy have previously been reported to significantly improve the median OS (15.9–22 months) and PFS (6.4–8.8 months) of patients with inoperable advanced NSCLC in different trials (KEYNOTE-024^72^, KEYNOTE-189^73^, and KEYNOTE-407^74^). Nevertheless, there are still several major problems in the immunotherapy of NSCLC^11,75^. The resistance to these agents remains an insurmountable challenge currently.

Although salvage surgery after ICIs is rarely reported, its frequency is increasing with the application of immunotherapy in NSCLC. Recently, a total of 164 advanced patients who underwent salvage surgery after immunotherapy were reported at the 103th annual meeting of the American Association for Thoracic Surgery^76^. The 30-, 90-day mortality and 3-year OS rates of salvage surgery were 0.6, 4.3, and 77%, respectively. In addition, another study^77^ reviewed 11 patients with advanced NSCLC who underwent salvage surgery after ICI therapy. The perioperative mortality and 90-day mortality were 0 and 9% (one patient died of acute exacerbation of interstitial pneumonia). In another study, salvage surgery after immunotherapy for previously metastatic tumors or unresectable NSCLC (47%) was reported in 19 cases^78^. The 2-year OS and disease-free survival rates reported in the whole patient cohort were 77 and 42%, respectively.

Safety and technical issues related to lung resection after ICI were reported, indicating that salvage surgery was feasible. The perioperative complication rate reported by Ueno *et al*.^77^ was 27%, which was consistent with previous research results^78^. The common complications were air leakage, arrhythmia, and respiratory tract infection. Technically, due to the side effects of immunotherapy, some patients develop severe pulmonary fibrosis. In patients with more advanced disease, infection and dense adhesions in the surrounding hilar and mediastinal lymph node stations increased the difficulty of surgery. Moreover, the potential toxicity characteristics of immunotherapy agents can pose specific concerns for salvage surgery^79^. Autoimmune toxicity, especially pneumonia, is a concern during the perioperative period.

## Conclusion

Conversion surgery is a potential and promising treatment for further improving the outcomes in patients with advanced NSCLC. However, previous evidence usually confused it with true salvage surgery and designed as a single-arm study. Currently, we lack strong evidence to confirm its effectiveness. Meanwhile, timing and accurate preoperative staging probably influence the effectiveness of conversion surgery. A comparative study with a rigorous study design is warranted to define the concept of conversion surgery clearly and confirm its survival benefit for advanced NSCLC. Salvage surgery is a safe and effective treatment for selected patients with residual or recurrent NSCLC. The effectiveness of salvage surgery is mainly based on the initial stage and treatment. For early-stage NSCLC after initial surgery or SBRT, salvage surgery could generally achieve good effectiveness. In contrast, the effectiveness of salvage surgery for advanced NSCLC varied with the initial stage, tumor response to initial systemic treatments, and treatment interval. Multidisciplinary evaluation is recommended for the decision of conversion surgery and salvage surgery.

## Ethical approval

Ethical review and approval were waived for this study due to the article being a review.

## Consent

Patient consent was not required due to the article being a review.

## Source of funding

Not applicable.

## Author contribution

H.-J.Y., X.-Y.Z., Y.-T.Z., W.-J.M., and D.T.: conceptualization; H.-J.Y., X.-Y.Z., and Y.-T.Z.: writing – original draft; J.-X.W., J.C., Z.-Q.D., Y.-Y.M., W.-L.H., J.-J.Z., A.-L.Z., W.-J.M., and D.T.: writing – review and editing; C.-Y.Z.: visualization.

## Conflicts of interest disclosure

The authors declare no conflicts of interest.

## Research registration unique identifying number (UIN)

1. Name of the registry: not applicable.
2. Unique Identifying number or registration ID: not applicable.
3. Hyperlink to your specific registration (must be publicly accessible and will be checked): not applicable.

## Guarantor

Dong Tian and Wen-Jun Mao.

## Data availability statement

Not applicable.

## Provenance and peer review

Not commissioned, externally peer-reviewed.
